# Strength of Carbolic Acid Solutions

**Published:** 1868-10

**Authors:** 


STRENGTH OF CARBOLIC ACID SOLUTIONS.—In view of the fact that carbolic acid is now largely in use in medicine, with a probability that its range of application will be increased, it is well for prescribers to be very careful of the particular preparation they employ. Instances are reported where much damage has been done by the external application of this substance in solution, the prescriber not knowing the exact strength of the solution, and we ourselves have seen carbolic acid ordered from the apothecaries, in such a way as to evince plainly the fact of a most blissful ignorance of whether the medicine was a solid or a fluid, or in what proportions it was proper to use. Dr. W. T. Channing, of Providence, reports to the Boston *Journal of Chemistry* several cases of serious results, from the use of the concentrated fluid acid, which is dispensed by some under the name of "solution carbolic acid," when the prescribers intended only a milder solution, which they had been in the habit of using, but had obtained it from other druggists. Until, therefore, some distinctive nomenclature shall be given to the various preparations of this substance, and some officinal "solution" shall be decided upon, physicians cannot be too careful in learning the strength of the solution employed, and it would be advisable to give explicit directions where to procure it.—*N. Y. Journal of Medicine.*

